# A lived experience transformation of mental illness to mental health: Inspiring a movement of hope

**DOI:** 10.1177/10398562251346609

**Published:** 2025-06-02

**Authors:** James McLure

**Affiliations:** School of Medicine, The Institute for Mental and Physical Health and Clinical Translation (IMPACT), 2104 Deakin University, Geelong, Australia

**Keywords:** transformation, hope, lived experience, personal testimony, mental health systems

## Abstract

**Objective:**

The objective is to reflect on my own lived experience to affirm the importance of hope in fuelling personal growth after an experience of mental illness. My personal testimony describes mental illness ultimately as a blessing as it precludes a humbling and exquisite transformation and healing process. Secondly, to provide a narrative review of the ways hope may spread through mental health systems, identifying peer support as a current example. Finally, to illustrate the importance of people with lived experience leading research in mental health as it may guide the field to areas of greatest impact.

**Conclusions:**

A narrative of lived experience testimony of mental illness and subsequent transformation can inspire hope and sustained growth in individuals and communities. One of the most important drivers of profound healing comes in the form of hope. Currently, peer support workers employed in mental health systems embrace companionship with the people accessing services while simultaneously growing to sounder mental health together through hopeful, healing relationships. Lived experience leadership in research also continues to grow and drives specific and unique insights into grant, protocol, and policy development.

In the *World Mental Health Report: Transforming Mental health for all,* the World Health Organization encourages nations to invest in mental health.^1^ Over 980 million people were reported to experience a diagnosable mental disorder globally.^1^ This statistic highlights an urgent need for investment into, and propagation of hope towards, global mental health. Reflecting on my own lived experience, mental illness was the catalyst which ultimately led to a hope-filled life-changing experience, a sustained new cycle of personal growth and transformation into a truer and improved version of myself. Such experiences are also reported in the literature.^2–5^

In the initial phase of personal growth and healing, post a seven-and-a-half-year experience of psychosis, hope was not something I encountered. During the first 9 months post hospitalization, the days were especially long and arduous, and my personal growth was excruciatingly slow. I vividly remember when I left an acute mental health ward in regional Victoria, the team at the time said, ‘you’ll be back’, I thought to myself ‘umm...no I won’t’. The team knew the illness that I had been diagnosed with, but not the person I am, the family I hail from, or the Supreme Healer who was at work in, and around, me. This is an example of the lack of hope that I experienced from the mental health system at the time.

A little further on in my journey, while going on a daily walk, I experienced one second of hope, in that moment I knew I could grow to mental health and happiness. Enter the complex interplay of enduring the illness and growing gradually, developing and maintaining relationships, returning to work, increasing conditioning daily, and nurturing this newfound hope like my life depended on it. With this experience of hope, my will for wellbeing was illumined. I committed to daily personal growth and healing and supporting others in doing similarly, the purpose and blessing my dual role as a peer support worker and mental health researcher now provides.

The suffering experienced through mental illness, and especially the initial period of connecting with sustained growth and healing can ultimately lead to spiritual integrity, character refinement, personal strengthening, deepened commitment to sound values, increased empathy for one-self and others, more loving relationships with family and friends, and a greater appreciation for life.^2,3,5,6^ This was true indeed in my own lived experience, and one of the principles which spurred close to two decades of sustained personal growth in me was GROW’s Principle of Hope: ‘*I can, and ultimately will, become completely well; God who made me can restore me and enable me to do my part. The best of life and love and happiness is ahead of me’*.^5, p.16^ GROW is an international mental health movement which originated in Sydney, in 1957. This movement utilizes a unique store of lived experience wisdom, attitudes, and insights of a global community of ‘Growers’ committed to obtaining mental health and happiness post mental decline. GROW essentially models a way of healing and ongoing growth and development for individuals and communities. The movement is based around connecting with ourselves, and others, and for those who believe in God, with Him also. There is a healing spirit of friendship which emanates. Notably, I refer to GROW’s Program of Personal Growth throughout this perspective piece, as its wisdom and philosophy transformed my life.^5^

## Propagating hope in mental health systems

Mental health systems are often the first port of call for those individuals most in need of support for mental illness. Mental health systems around the world are however often under-resourced and over-stretched to meet the specific needs of people experiencing mental illness and transforming towards mental health.^1,7^ The State of Victoria, Australia, presents an example of the need to reform a mental health system; where the Royal Commission into Victoria’s Mental Health System exposed painful truths through the personal testimonies of thousands of people with lived experience of the system, some not unlike mine.^8^ The cumulative wisdom and experience of these respondents was used to formulate a remedy for an ailing mental health system. Four years on since the Royal Commission into Victoria’s Mental Health System Final Report and the state of Victoria’s public mental health system is undergoing a radical transformation.^8^ By embracing lived experience leadership, together with highly significant investment in infrastructure and staffing of mental health and wellbeing units and hubs, new governance and accountability structures, and a new *Mental Health and Wellbeing Act, 2022*, Victoria’s Mental Health System has improved significantly.^9^

In creating conditions conducive to transformation, individuals and communities require mental health systems and policy that understand their needs. The World Health Organization and the State of Victoria recommend person-centred recovery-oriented system design approaches with input from those who engage in mental health systems and whom can share their lived experience.^1,10^ Notably, there is a research team within Deakin University who offer experienced facilitation of a novel co-design methodology using group model building, tailored specifically to elevate the voice of lived experience in mental health research.^11,12^

Through offering peer support in an acute mental health facility I have learnt that individuals accessing acute mental healthcare often experience deep personal challenge while simultaneously finding themselves primed for personal change. Mental health systems need not miss the opportunity to support transformation in the individuals they support. Transformation can be facilitated, for a start, by expressing genuine empathy and compassion for an individual’s or community’s struggles, holding hope for, and retaining the dignity of, people impacted by mental illness, using person-centred growth-oriented language and care, and essentially by loving people back to health.^2,5^

One of the most recent revolutionary service delivery offerings in public mental health services is that of peer support. This movement is spreading rapidly around the world, for it is companionship, empathy, shared growth towards mental health, lived experience leadership, and living hope for people traversing incredibly challenging transformations.^13,14^ The origins of peer support in mental health services date back over 230 years.^15^ A mental health physician in France, Philippe Pinel, identified that ex-patients of the mental health system were gentle, humane, and disposed to kindness and were highly effective in playing a part in the healing of mentally unwell individuals.^15,16^ Profoundly, these attributes hold true for peer workers today, as they continue to courageously meet individuals with extreme mental health challenges where they are at. Furthermore, the peer workers I’ve mentored are deeply grateful for the peer relationships they form, connecting in a wholesome and heartfelt manner while embracing a shared maturity.

All workers in the mental health system need to be adept transformation specialists, ideally growing into greater humanity themselves, and able to provide companionship with depth, together with their discipline specific skills.^2^ Multi-disciplinary teams are important to holistically respond to the breadth of challenge and debilitation that exists for people affected by mental illness. A recent paper by Berk et al.^17^ describes multi-disciplinary teams emphasizing small gains in people’s growth in areas such as hobbies, socializing, working, and exercising as vitally important in improving mental and physical health.^17^ In the early stage of transforming, in attempting to correct bad habits of thinking, feeling, and living, ‘*growth is painful but permanently rewarding*’.^5, p.30^ Enormous effort needs to continue to be invested into the people accessing services, as in the overall plan, these people may be the free and gentle builders of the community in which they live.^5^ And ultimately, when people are personally growing and healing, GROW’s Principle of Universal Benefit applies: ‘*Each person’s recovery or growth aids the transformation of the world’*.^5, p.84^

Mental health systems around the world requiring improvement can learn from the State of Victoria, Australia’s system change.^1,8^ In the interim, prioritizing raising awareness of the evidence of transformation at the individual and community level is vital for the momentum of this mental health movement.^4,7^ Every person has a part to play. In supporting personal growth and healing in individuals and communities we want to ensure no person misses the opportunity to meet with friendly, empathic, and humane supporters who understand their needs and support the emergence of the new and true self.^5^
